# Prognostic significance of serum inflammatory markers in esophageal cancer

**DOI:** 10.1007/s10388-020-00772-3

**Published:** 2020-08-31

**Authors:** Arfon G. M. T. Powell, Catherine Eley, Carven Chin, Alexandra H Coxon, Adam Christian, Wyn G. Lewis, South East Wales Oesophagogastric Cancer Collaborative

**Affiliations:** 1 Division of Cancer and Genetics, University Hospital of Wales, Cardiff University, Heath Park, Cardiff, UK; 2 Department of Surgery, Cardiff & Vale University Health Board, University Hospital of Wales, Heath Park, Cardiff, UK; 3 Department of Pathology, Cardiff & Vale University Health Board, University Hospital of Wales, Heath Park, Cardiff, UK

**Keywords:** Esophageal cancer, Systemic inflammation, Survival

## Abstract

**Background:**

The aim of this study was to assess the relative prognostic value of biomarkers to measure the systemic inflammatory response (SIR) and potentially improve prognostic modeling in patients undergoing potentially curative surgery for esophageal adenocarcinoma (EC).

**Methods:**

Consecutive 330 patients undergoing surgery for EC between 2004 and 2018 within a regional UK cancer network were identified. Serum measurements of haemoglobin, C-reactive protein, albumin, modified Glasgow Prognostic Score (mGPS), and differential neutrophil to lymphocyte ratio (NLR) were obtained before surgery, and correlated with histopathological factors and outcomes. Primary outcome measures were disease-free (DFS) and overall survival (OS).

**Results:**

Of 330 OC patients, 294 underwent potentially curative esophagectomy. Univariable DFS analysis revealed pT, pN, pTNM stage (all *p* < 0.001), poor differentiation (*p* = 0.001), vascular invasion (*p* < 0.001), R1 status (*p* < 0.001), perioperative chemotherapy (*p* = 0.009), CRP (*p* = 0.010), mGPS (*p* = 0.011), and NLR (*p* < 0.001), were all associated with poor survival. Multivariable Cox regression analysis of DFS revealed only NLR [Hazard Ratio (HR) 3.63, 95% Confidence Interval (CI) 2.11–6.24, *p* < 0.001] retained significance. Multivariable Cox regression analysis of OS revealed similar findings: NLR [HR 2.66, (95% CI 1.58–4.50), *p* < 0.001].

**Conclusion:**

NLR is an important SIR prognostic biomarker associated with DFS and OS in EC.

## Introduction

Biomarkers, at their best, deliver data in three important domains. First, to help diagnose conditions (identifying early stage cancers—diagnostic); second, to forecast aggressive conditions (prognostic); and third, to predict how well a patient will respond to treatment (predictive) [1].

Esophageal cancer (EC) is the sixth leading worldwide cause of cancer related death, accounting for some half a million deaths annually [2]. Surgery remains the only potentially curative treatment, yet almost half of patients develop recurrence, and adjuvant therapies, including chemotherapy or chemoradiotherapy lack global consensus, with no established standard of care [3]. Cancer-related inflammation has been dubbed the 7th hallmark of cancer [4], and the systemic inflammatory response (SIR) is measured using cellular (whole white cell counts, neutrophils, lymphocytes, and platelets), and humoral (C-reactive protein (CRP) and albumin) components. Derivative biomarkers neutrophil–lymphocyte ratio (NLR), platelet-lymphocyte ratio (PLR), neutrophil-platelet score (NPS), and the modified Glasgow Prognostic Score (mGPS), have also been reported to be associated with poor survival [5–7]. If SIR is to become a therapeutic target then a single, sensitive, specific, and reproducible marker is essential, but so far, although multivariable regression models have incorporated common clinic-pathological factors, no study has scrutinized the relative prognostic significance of SIR derived biomarkers in EC.

The aim of this study was to determine if a single biomarker of SIR was independently associated with survival, after potentially curative esophagectomy for cancer. The hypothesis was that a composite biomarker of SIR would have independent significant prognostic value, regardless of histopathological TNM stage, and other SIR biomarkers on multivariable regression modeling. The setting was a regional UK cancer network serving a population of 1.8 million.

## Methods

### Patients

In order to test the hypothesis a single cohort was developed including patients of radiological TNM stage I to III, deemed to have potentially curable esophageal adenocarcinoma, between January 2004 and August 2018, and treated by a cancer network specialist multidisciplinary team, serving a population of 1.8 million. All patients had management plans individually tailored according to factors related to both patient and disease. Staging was by means of computed tomography, endoscopic ultrasound, computed tomography positron emission tomography, and staging laparoscopy as appropriate [8]. The network MDT treatment algorithms for EC have been described previously [9].

The standard operative approach of subtotal Trans Thoracic esophagectomy (TTO) as described by Lewis [10] and Trans Hiatal esophagectomy (THO), as described by Orringer [11], was used selectively in patients with adenocarcinoma of the lower third of the oesophagus who had significant cardiorespiratory co-morbidity, cT1/2 cN0 or cT3 cN0 disease. A modified extended D2 lymphadenectomy (preserving pancreas and spleen where possible) was performed in all cases. Sixteen patients underwent laparoscopic assisted surgery during the study period. All patients received an Enhanced Recovery After Surgery (ERAS) programme as described previously [12, 13]. Fit patients with tumours of stage cT3 or equivocal cT4 and cN0 or any cT and cN1 were treated with neoadjuvant therapy before surgery [13]. The majority of these patients received 2 cycles of 80 mg/m^2^ of Cisplatin and 1000 mg/m^2^ of 5-Fu for 4 days. A minority received 4 cycles of Epirubicin (50 mg/m^2^), Cisplatin (60 mg/m^2^) and 5-Fu (200 mg/m^2^) or Capecitabine (625 mg/m^2^; ECF/X).

Ethical approval was sought, but the chair of Cardiff & Value University Health Board ethics committee confirmed that individual patient consent was not required to report clinical outcomes alone, and no formal approval was necessary.

### Clinicopathological characteristics

Tumours were staged using the seventh edition of the AJCC/UICC-TNM staging system. Pathological factors were recorded from reports issued at the time of surgery and included tumour differentiation, vascular invasion, margin status, and the number of lymph nodes with and without metastasis.

Laboratory whole white-cell count, neutrophil count, lymphocyte count, platelet counts, CRP, and albumin prior to surgery were recorded as described by others [14, 15]. Derivate measurements of the SIR consisted of NLR, NPS, PLR and the mGPS were calculated. These derivative measurements were dichotomised into low and high groups by 2.5 for NLR, and 150 for PLR [6]. The NPS was constructed by grouping patients into three cohorts; zero (0) for patients with both normal neutrophil (≤ 7.5 × 10^9^/L) and platelet counts (≤ 400 × 10^9^/L), one (1) for patients with either a high neutrophil (> 7.5 × 10^9^/L) or platelet count (> 400 × 10^9^/L), and two (2) for patients with both high neutrophil and platelet count. The mGPS was constructed using CRP and albumin. Patients with normal serum levels of CRP (≤ 10 mg/l) and albumin (≥ 35 g/l) were given a score of zero. Patients with a raised serum CRP (> 10 mg/l) and normal serum albumin were given a score of one, and patients with a raised serum CRP and low serum albumin (< 35 g/l) were given a score of two [16].

Patients were followed up at regular intervals of 3 months for the first year and 6 months thereafter. In the event that patients developed symptoms suggestive of recurrent disease, investigations were undertaken sooner. The follow-up surveillance was conducted for 5 years or until death. Death certification was obtained from the Office for National Statistics via Cancer Network Information System Cymru (CaNISC).

### Statistical analysis

Sample size calculations were based on a pre-study literature survey of (CRUK cancers statistics [17]), which indicated that the baseline five-year survival rate of patients diagnosed with stage II EC was expected to be 40%, compared with 20% in patients with stage III EC, and a 15% difference in survival would be a realistic expectation. Thus, a minimum of 276 patients were to be studied, providing 80% power to detect such a difference with alpha set at *p* < 0.05.

Grouped data were expressed as median (range) and non-parametric methods used throughout. Disease-free survival for all patients was calculated by measuring the interval from a landmark time of 6 months after diagnosis to the date of recurrence. This approach was adopted in previous randomized trials [18], to allow for the variable interval to surgery following diagnosis, depending on whether neoadjuvant therapy was prescribed. As in these trials, events resulting in a failure to complete curative treatment, such as not proceeding to surgery, open and close laparotomy, palliative resection, in-hospital mortality and disease progression during neoadjuvant chemotherapy, were assumed to have occurred at this landmark time, to maintain the intention-to-treat analysis. Overall survival was measured from the date of diagnosis. Cumulative survival was calculated according to the Kaplan–Meier method; differences between groups were analyzed with the log rank test. Univariable analyses examining factors influencing survival were examined initially by the life table Kaplan–Meier method, and those with associations found to be significant on log-rank analysis (*p* < 0·100) were retained in a Cox proportional hazards model using forward conditional methodology to assess the prognostic value of individual variables. All statistical analysis was performed in SPSS^®^ (IBM^®^ SPSS^®^ Statistics v25.0.0.0, IBM Corporation, Armonk, New York, USA) with extension R.

## Results

### Patients, clinico-pathological factors and features associated with non-resectability

In total, 330 patients were identified who underwent surgery for EC. Thirty-six patients (10.9%) were deemed to have inoperable tumours because of local invasion. The remaining 294 patients underwent potentially curative esophagectomy. The patient cohort undergoing palliative surgery had raised serum CRP measurements (41.7% vs. 16.0%, *p* < 0.001), hypoalbuminaemia (36.1% vs. 16.7%, *p* = 0.005), higher mGPS (27.8% vs. 6.6%, *p* < 0.001), thrombophilia (13.9% vs. 2.7%, *p* = 0.001), higher NPS (2.8% vs 0.3%, *p* = 0.006), and were more likely to have received neoadjuvant chemotherapy (91.4% vs. 70.4%, *p* = 0.008). On multivariable binary logistical regression analysis of factors associated with poor survival on univariable analysis, mGPS (Odds Ratio (OR) 2.29 (95% Confidence Interval (95% CI) 1.44–3.62), *p* < 0.001), thrombophilia (OR 4.76 (1.26–18.06), *p* = 0.022) and neoadjuvant therapy (OR 3.72 (1.10–11.57), *p* = 0.023) were independently associated with inoperability. The area under the curve (AUC) for neoadjuvant therapy was 0.59 (95% CI 0.50–0.68, *p* = 0.079), AUC for thrombophilia 0.59 (95% CI 0.45–0.67, *p* = 0.263), and AUC for mGPS 0.64 (95% CI 0.54–0.75, *p* = 0.006).

### Details of the patients undergoing potentially curative esophagectomy

The characteristics of clinico-pathological variables studied can be found in Tables 1 and 2. The median age for patients undergoing resection was 69 years (inter-quartile range (IQR) 62–74) with the majority (*n* = 141, 48.0%) being between 65 and 75 years of age (Table 2). Most patients were male (*n* = 250, 85.0%), and were lymph node positive (*n* = 149, 50.7%). Perioperative chemotherapy was prescribed in 207 patients (70.4%, Tables 1 and 2). During follow-up, 86 patients (29.2%) developed cancer recurrence and 106 patients (36.1%) died. Median follow-up of the surviving patients was 34 (range 6–60) months and 194 (58.8%) of patients were followed-up for 5 years or until death.

**Table 1:** The relationship between tumour related factors, overall and disease-free survival in patients undergoing potentially curative resection for esophageal cancer

		Disease-free survival		Overall survival	
Clinicopathological variables	Frequency *n* (%)	5-year survival rate (%)	*p*-value	5-year survival rate (%)	*p*-value
T stage					
CR	22 (7.5)	100.0	< 0.001	100.0	< 0.001
1	74 (25.2)	84.0		80.0	
2	32 (10.9)	45.5		40.9	
3	144 (49.0)	37.0		33.8	
4	22 (7.5)	0.0		0.0	
N stage					
0	145 (49.3)	72.9	< 0.001	69.5	< 0.001
1	80 (27.2)	35.6		31.1	
2	48 (16.3)	11.1		5.6	
3	21 (7.1)	11.1		11.1	
Tumour Stage					
CR	22 (7.5)	100.0	< 0.001	100.0	< 0.001
I	82 (27.9)	72.7		69.7	
II	60 (20.4)	63.9		58.3	
III	130 (44.2)	20.3		16.9	
Differentiation					
Well/Moderate	175 (59.5)	58.0	0.002	53.1	0.005
Poor	119 (40.5)	30.0		28.0	
Vascular invasion					
No	153 (52.0)	68.8	< 0.001	67.2	< 0.001
Yes	141 (48.0)	26.9		20.9	
Lymph node sample					
≥ 15	154 (52.4)	48.8	0.667	45.1	0.631
< 15	140 (47.6)	44.9		40.8	
R status					
0	179 (60.9)	62.2	< 0.001	55.4	0.002
1	115 (39.1)	28.1		28.1	
Perioperative chemotherapy					
No	87 (39.6)	65.8	0.007	65.8	0.001
Yes	207 (70.4)	39.8		34.4	
					0

**Table 2:** The relationship between patient related factors, overall survival and disease-free survival in patients undergoing potentially curative resection for esophageal cancer

		Disease-free survival		Overall survival	
Clinico-pathological variables	Frequency *n* (%)	5-Year survival rate (%)	*p*-value	5-year survival rate (%)	*p*-value
Age (years)					0.991
< 65	100 (34.0)	47.2	0.581	44.4	
65–75	141 (48.0)	43.1		43.1	
> 75 years	53 (18.0)	54.1		43.2	
Sex					0.714
Female	44 (15.0)	52.6	0.617	47.4	
Male	250 (85.0)	46.4		42.9	
White Cell count					0.876
Low	20 (6.8)	50.0	0.878	50.0	
Normal	269 (91.5)	47.5		43.3	
High	5 (1.7)	33.3		33.3	
Neutrophil count					0.743
Low	280 (95.2)	47.2	0.893	43.2	
High	14 (4.8)	50.0		50.0	
Lymphocyte count					0.501
Low	44 (15.0)	47.1	0.623	41.2	
Normal	242 (82.3)	46.3		42.6	
High	8 (2.7)	66.7		66.7	
Platelet count					0.042
Low	286 (97.3)	46.1	0.065	42.2	
High	8 (2.7)	100.0		100.0	
C-Reactive Protein					0.585
Normal	247 (84.0)	49.1	0.355	44.5	
High	47 (16.0)	38.1		38.1	
Albumin					0.984
Normal	245 (83.3)	47.8	0.760	43.5	
Low	49 (16.7)	43.8		43.8	
Derivative markers					
Neutrophil–Lymphocyte Ratio					0.137
Low	263 (89.5)	45.3	0.233	49.6	
High	31 (10.5)	28.6		28.6	
Neutrophil–Platelet Score					0.147
Low	273 (92.9)	45.9	0.229	41.8	
Intermediate	20 (6.8)	66.7		66.7	
High	1 (0.3)	N/A		N/A	
Platelet–Lymphocyte Ratio					0.765
Low	137 (46.6)	48.4	0.804	42.2	
High	157 (53.4)	46.3		44.8	
Modified Glasgow prognostic Score					0.593
Low	247 (84.0)	49.1	0.452	44.5	
Intermediate	28 (9.5)	30.8		30.8	
High	19 (6.5)	50.0		50.0	

### Relationships between the SIR, clinico-pathological factors and survival

Univariable and multivariable analyses of the factors associated with both disease-free and overall survival can be found in Table 3. The number of events per variable was 10.6. The relationship between NLR and clinico-pathological factors is shown in Table 4.

**Table 3:** Univariable and multivariable analyses of clinico-pathological factors and serum inflammatory markers; disease-free and overall survival

	Univariable		Multivariable		Univariable		Multivariable	
	Disease-free survival				Overall survival			
	Hazard ratio (95% CI)	*p*-value	Hazard ratio (95% CI)	*p*-value	Hazard ratio (95% CI)	*p*-value	Hazard ratio (95% CI)	p-value
Age (< 65/65–75 / > 75)	1.05 (0.78–1.41)	0.762			0.92 (0.71–1.20)	0.530		
Gender (female/male)	1.09 (0.58–2.05)	0.792			1.10 (0.63–1.93)	0.742		
pT stage (1/2/3/4)	2.01 (1.56–2.58)	< 0.001		0.522	2.23 (1.74–2.86)	< 0.001		0.267
pN stage (0/1/2/3)	2.07 (1.69–2.54)	< 0.001		0.066	2.20 (1.84–2.63)	< 0.001	1.67 (1.33–2.10)	< 0.001
pTNM stage (I/II/III)	2.63 (1.96–3.54)	< 0.001	1.51 (1.00–2.29)	0.051	2.75 (2.09–3.63)	< 0.001		0.362
Differentiation (well/moderate/poor)	2.21 (1.44–3.39)	< 0.001		0.182	2.59 (1.76–3.81)	< 0.001	1.58 (1.05–2.36)	0.027
Vascular invasion (no/yes)	5.26 (3.22–8.60)	< 0.001	3.03 (1.75–5.26)	< 0.001	4.60 (2.98–7.11)	< 0.001	2.87 (1.82–4.52)	< 0.001
R status (0/1)	2.49 (1.62–3.82)	< 0.001		0.574	3.29 (2.34–4.86)	< 0.001		0.059
Peri-operative chemotherapy (no/yes)	2.00 (1.19–3.38)	0.009		0.073	1.66 (1.06–2.61)	0.027		0.111
C-reactive protein (normal/high)	1.94 (1.17–3.20)	0.010		0.504	1.94 (1.23–3.06)	0.004		0.363
mGPS (0/1/2)	1.50 (1.10–2.05)	0.011		0.839	1.48 (1.11–1.98)	0.008		0.815
NLR (low/high)	3.08 (1.81–5.24)	< 0.001	3.63 (2.11–6.24)	< 0.001	2.39 (1.44–3.97)	0.001	2.66 (1.58–4.50)	< 0.001

**Table 4:** The relationship between NLR and clinico-pathological factors in patients undergoing potentially curative resection for esophageal cancer

Clinico-pathological factors	NLR low *n* (%)	NLR high *n* (%)	*p*-value
Age (years)			0.538
< 65	88 (33.5)	12 (38.7)	
65–75	129 (49.0)	12 (38.7)	
> 75 years	46 (17.5)	7 (22.6)	
Sex			0.848
Female	39 (14.8)	5 (16.1)	
Male	224 (85.2)	26 (83.9)	
T stage			0.150
CR	17 (6.5)	5 (16.1)	
1	70 (26.6)	4 (12.9)	
2	28 (10.6)	4 (12.9)	
3	127 (48.3)	17 (54.8)	
4	21 (8.0)	1 (3.2)	
N stage			0.993
0	129 (49.0)	16 (51.6)	
1	72 (27.4)	8 (25.8)	
2	43 (16.3)	5 (16.1)	
3	19 (7.2)	2 (6.5)	
Tumour stage			0.213
CR	17 (6.5)	5 (16.1)	
I	76 (28.9)	6 (19.4)	
II	53 (20.2)	7 (22.6)	
III	117 (44.5)	13 (41.9)	
Differentiation			0.549
Well/moderate	155 (58.9)	20 (64.5)	
Poor	108 (41.1)	11 (35.5)	
Vascular invasion			0.960
No	137 (52.1)	16 (51.6)	
Yes	126 (47.9)	15 (48.4)	
Lymph node sample			0.018
≥ 15	144 (54.8)	10 (32.3)	
< 15	119 (45.2)	21 (67.7)	
R status			0.466
0	162 (61.6)	17 (54.8)	
1	101 (38.4)	14 (45.2)	
Peri-operative chemotherapy			0.031
No	83 (31.6)	4 (12.9)	
Yes	180 (68.4)	27 (87.1)	
White cell count			< 0.001
Low	18 (6.8)	2 (6.5)	
Normal	244 (92.8)	25 (80.6)	
High	1 (0.4)	4 (12.9)	
Neutrophil count			< 0.001
Low	261 (99.2)	19 (61.3)	
High	2 (0.8)	12 (38.7)	
Lymphocyte count			< 0.001
Low	24 (9.1)	20 (64.5)	
Normal	231 (87.8)	11 (35.5)	
High	8 (3.0)	0 (0.0)	
Platelet count			0.177
Low	257 (97.7)	29 (93.5)	
High	6 (2.3)	2 (6.5)	
CRP			< 0.001
Normal	230 (87.5)	17 (54.8)	
High	33 (12.5)	14 (45.2)	
Albumin			< 0.001
Normal	227 (86.3)	18 (58.1)	
Low	36 (13.7)	13 (41.9)	
Modified Glasgow Prognostic Score			< 0.001
0	230 (87.5)	17 (54.8)	
1	23 (8.7)	5 (16.1)	
2	10 (3.8)	9 (29.0)	
Neutrophil-Platelet Score			< 0.001
Low	255 (97.0)	18 (58.1)	
Intermediate	8 (3.0)	12 (38.7)	
High	0 (0.0)	1 (3.2)	
Platelet-Lymphocyte ratio			< 0.001
Low	136 (51.7)	1 (3.2)	
High	127 (48.3)	30 (96.8)	

## Discussion

The principal finding of this study was that NLR emerged as the only significant inflammatory prognostic biomarker, in a cohort of UK patients with EC, supporting the primary hypothesis. No fewer than one in six patients had raised SIR markers, and some one in ten a raised NLR. Patients with mGPS of two were nearly five times more likely to have inoperable cancers when compared with patients with mGPS of zero. Moreover, elevated NLR, CRP, and mGPS, were all associated with poorer disease-free and overall survival profiles. Patients with a low NLR experienced median DFS and OS, on average 18 and 13 months respectively better than patients with a high NLR. Similarly, patients with a low NLR experienced five-year DFS and OS of 45.3%, and 49.6%, 1.6 and 1.8 fold better than patients with a high NLR.

The inflammatory markers described in this study can be broadly characterized as hepatic (mGPS and its components) or haematological (NLR, NPS, PLR, and their components), based on their predominant area of activity. NLR and mGPS have been associated with poor survival in a raft of anatomical cancer sites including breast [19, 20], colorectal [21, 22], stomach [6, 23], and prostate [24, 25]. In gastric [6], colorectal [22], and prostate [24] cancer patients with a mGPS of two had a five-year survival of 20%, 45%, and 33%, compared with 80%, 85%, and 75% in paients with mGPS of zero respectively. With regard to mGPS and NLR, 16.5% and 10.6% of patients respectively, had evidence of SIR on pre-operative blood analysis. This compares with previous reports citing SIR of 20% (mGPS), and 10% (NLR) of gastric cancer patients [6], and 41% [26] (mGPS) and 19% (NLR) of colorectal cancer patients [5]. But in contrast to reports in gastric and colorectal cancer, NLR was the only prognostic inflammatory biomarker found to be associated with survival in EC. Cumulative 5-year differential survival related to NLR expression have been reported to be similar in both esophageal (17%) and colorectal cancer (20%) [5]. Yet the absolute 5-year survival in the patient cohorts with low NLR expression was 45% in esophageal, compared with 75% in colorectal cancer [5]; arguably consistent with the more aggressive clinical nature of EC when compared with colorectal cancer.

**Fig. 1:**
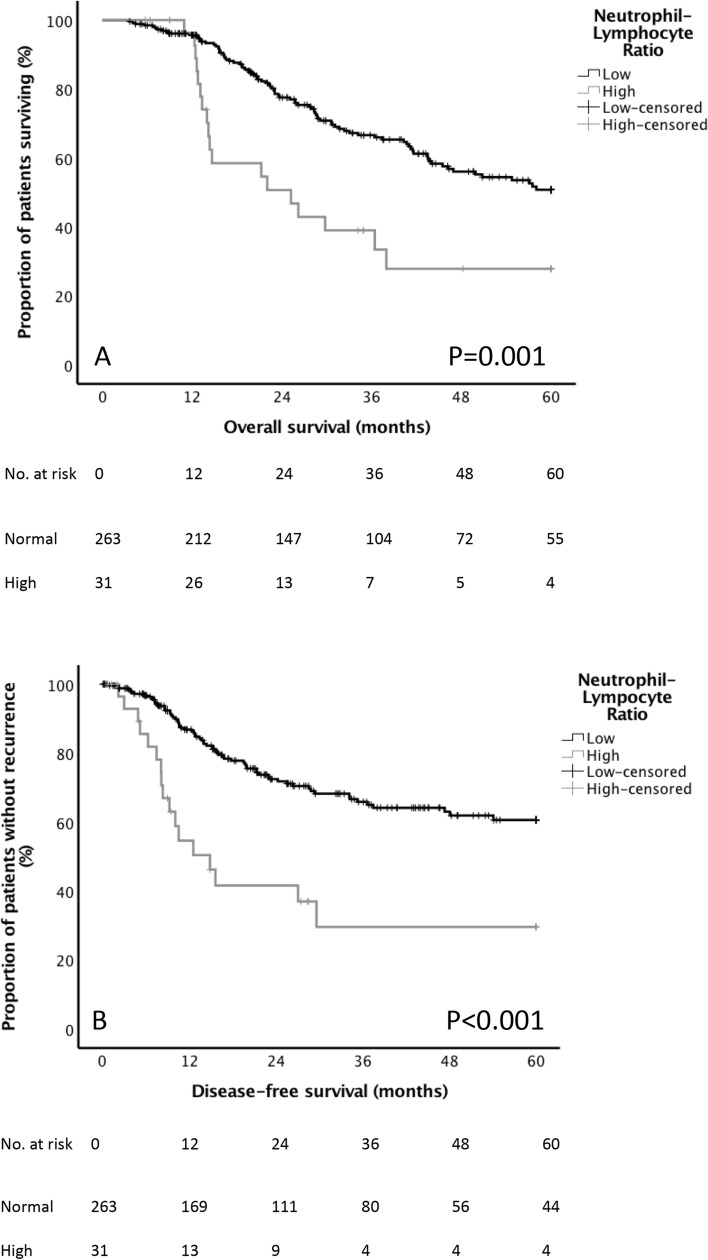
The relationship between NLR and survival; disease-free (**a**) and overall survival (**b**)

The underlying basis of the relationship between the SIR and poor cancer survival in patients with EC is an enigma. But the components of the mGPS have been characterized, with cancer cachexia, compromised cellular immune response, angiogenesis, and the up-regulation of growth factors all prominent features. It is evident therefore that the mechanisms controlling the association between systemic inflammation and cancer-specific survival are compound. Immune dysfunction, growth and dissemination, nutritional and functional decline, and tumour angiogenesis, are all implicated. More explicitly, recent colorectal cancer research has indicated a strong relationship between suboptimal patient physiological stage, increased comorbidity, and elevated CRP. In patients with an Eastern Cooperative Oncology Group Performance Status (ECOG-PS) zero or one, the median CRP was 20 mg/dl, compared with 64 mg/dl in the ECOG-PS of two (*p* = 0.002). Arguably, a raised preoperative mGPS in EC resonates with poor cardiorespiratory fitness, more comorbidity, and higher risk profile [27]. Most nutrition research professionals presently hold the view that in patients with cancer, nutritional status is closely related to the presence of a systemic inflammatory response as supported by the GPS, NLR, PLR, and NPS [28]. Indeed, cachexia is now considered by a number of expert nutrition groups to constitute disease related malnutrition in addition to inflammation, and comprises an etiologic factor in the GlIM criteria [29]. Future research should not only assess nutritional status but also the systemic inflammatory status in patients with EC cancer. The adverse prognostic power of the SIR is likely multifactorial in nature representing a more aggressive cancer phenotype on a background of a suppressed host response. However, this would be difficult to prove using association studies alone. Nevertheless, the data here confirms that survival following cancer is largely determines by the TNM stage, vascular invasion, and the presence of a SIR (Fig. 1).

Although SIR biomarkers offer valuable prognostic signals, if NLR and mGPS are to be incorporated into an upgraded TNM staging system, they should add prognostic value related to treatment response. Inclusion of biomarkers into gastric and breast cancer management algorithms was driven by the identification of adjuvant therapies for higher risk patients. Apart from Herceptin treatment for advanced esophago-gastric cancer, the principal adjuvant treatment for EC remains cytotoxic chemotherapy, despite pathological response rates of the order of one in seven [30]. Reports regarding the treatment of colorectal cancer with neoadjuvant [31, 32] or adjuvant chemotherapy [33] describe poorer outcomes in patients with SIR when compared with controls. Moreover, based on histological assessment, mGPS in rectal cancer has been reported to be associated with significantly poorer response to neoadjuvant chemotherapy [32]. Powell et al. from Cardiff, have also reported that an elevated pre-treatment NLR was associated with poorer tumour regression grade in patients undergoing neoadjuvant chemotherapy for EC [34]. Given the associations between the SIR and relative chemo-resistance, it is unlikely that such patients will derive any discernable clinical benefit from adjuvant chemotherapy. The phase III, double blinded, placebo controlled randomized trial regarding the effect of aspirin on disease recurrence and survival after primary therapy in non-metastatic solid tumours (Add-Aspirin), commenced recruitment in 2015, and should signal whether Aspirin is beneficial in EC treatment; yet arguably only patients with a SIR will respond. In keeping with other adjuvant treatments such as Herceptin and Cetuximab, patient selection is crucial and NLR has predictive biomarker potential in this regard.

There are a number of potential inherent limitations to studies of this character, which have been described previously [6, 35]. Cohort sample size was modest and study power was built on a 15% survival difference; sub-analysis related to patient comorbid risk profile, tumour stage, and morbidity severity-score was therefore not practical, and the above are therefore clearly confounding factors. Validating the results in an appropriately powered cohort for sub-analysis stage-for-stage, may facilitate the integration of NLR into a modified TNM staging system [36]. Though several SIR related bio-markers were used, the NLR may not predict survival independently in other patient cohorts and this matter needs international authentication. In contrast, the study has strengths. Patients were recruited from a consecutive patient cohort diagnosed with EC, from a single UK geographical region, all treated by a specialist multi-disciplinary team with standardized stage tailored treatment algorithms, operative techniques, with international peer-reviewed and published key performance indicator quality control. The survival data is particularly strong; no patients were lost to follow-up, and causes and dates of death were obtained from the office of national statistics. Moreover, the study builds on previous research by including all clinically available inflammatory and pathological factors in a multivariable regression model, and adds to the established evidence base, regarding the prognostic significance of preoperative SIR.

In summary, NLR was the sole inflammatory based prognostic biomarker associated with poor DFS and OS after potentially curative esophagectomy for cancer and was independent of histopathological stage. These findings emphasise the importance of not only staging the patient’s tumour radiologically; in essence a perceived histopathological stage; but also staging the patient’s physiological state using derivative SIR biomarkers. The results of this study consolidate the prognostic value of combined markers of the SIR, with the mGPS predicting patients at risk of inoperable disease at the time of surgery. This simple blood profile work deserves further considered reflection and should form part of routine preoperative patient work-up, and follow-up, for all such patients undergoing resection for cancer. These findings suggest that SIR presents narrative remedial goals, for patients vulnerable to cancer recurrence, and NLR may be the best biomarker to guide tailored holistic anti-inflammatory therapy, but further mechanistic studies are needed.

## Data Availability

We will not be making the dataset available, however, are more than happy to answer any questions of offer further information as required.
