# Correction to: Prognostic significance of serum inflammatory markers in esophageal cancer

**DOI:** 10.1007/s10388-021-00836-y

**Published:** 2021-04-12

**Authors:** Arfon G. M. T. Powell, Catherine Eley, Carven Chin, Alexandra H. Coxon, Adam Christian, Wyn G. Lewis, South East Wales Oesophagogastric Cancer Collaborative

**Affiliations:** 1 Division of Cancer and Genetics, University Hospital of Wales, Cardiff University, Heath Park, Cardiff, UK; 2 Department of Surgery, Cardiff & Vale University Health Board, University Hospital of Wales, Heath Park, Cardiff, UK; 3 Department of Pathology, Cardiff & Vale University Health Board, University Hospital of Wales, Heath Park, Cardiff, UK

**Correction to: Esophagus (2021) 18:267–277** 10.1007/s10388-020-00772-3

In the original publication of the article, under the section “Clinicopathological characteristics”, the thresholds used for NLR was published incorrectly as 2.5. The correct value is 5.5.

The correct sentence should read as follows, “These derivative measurements were dichotomised into low and high groups by 5.5 for NLR, and 150 for PLR [6]”.

